# Inter-eye relationship of intraocular pressure change after unilateral trabeculectomy, filtering canaloplasty, or PreserFlo™ microshunt implantation

**DOI:** 10.1007/s00417-021-05188-y

**Published:** 2021-05-08

**Authors:** Fidan A. Aghayeva, Panagiotis Chronopoulos, Alexander K. Schuster, Norbert Pfeiffer, Esther M. Hoffmann

**Affiliations:** 1 Department of Ophthalmology, University Medical Center of the Johannes Gutenberg University Mainz, Langenbeckstraße 1, 55131 Mainz, Germany; 2 National Centre of Ophthalmology named after academician Zarifa Aliyeva, Baku, Azerbaijan

**Keywords:** Fellow eye, Filtering canaloplasty, Glaucoma surgery, Intraocular pressure, PreserFlo™ microshunt, Trabeculectomy

## Abstract

**Purpose:**

This study assesses short-term intraocular pressure (IOP) change in the fellow eye of glaucoma patients after mitomycin C–augmented trabeculectomy, filtering canaloplasty, or PreserFlo™ microshunt implantation in the treated eye.

**Materials and methods:**

Retrospective chart review of 235 glaucoma patients (235 eyes) was performed. Patients underwent initial trabeculectomy (187 patients), filtering canaloplasty (25 patients), or PreserFlo™ microshunt implantation (23 patients) in one eye, while the fellow eye was naïve to any previous glaucoma surgery. IOP was evaluated before and on the 1st and 2nd days and at 1 week after surgery. Main outcome measure was IOP change in the fellow eye. Secondary outcomes were proportion of clinically significant IOP elevation in the fellow eye and evaluation of potential risk factors associated with postoperative IOP fluctuation.

**Results:**

IOP in the fellow eye at 1 week after trabeculectomy was statistically significantly lower than preoperatively (*p* < 0.0001), while the IOP did not change significantly in the fellow eyes in filtering canaloplasty or PreserFlo groups. The higher the preoperative IOP was in the fellow eye, the larger was the intraocular pressure-lowering effect at 1 week after trabeculectomy (*p* < 0.0001). A clinically significant IOP elevation was noted in 14.2%, 9.5%, and 5% of fellow eyes after trabeculectomy, filtering canaloplasty, or PreserFlo™ microshunt implantation*,* respectively.

**Conclusions:**

This study shows an IOP-lowering effect in the fellow eye of glaucoma patients after trabeculectomy. Significant IOP rise might occur in the fellow eye of some glaucoma patients after different types of glaucoma surgery.

**Supplementary Information:**

The online version contains supplementary material available at 10.1007/s00417-021-05188-y.

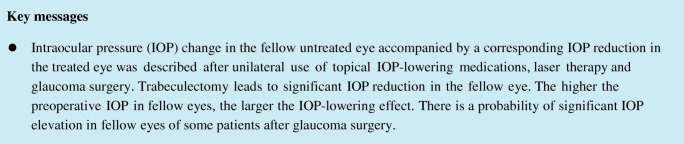

## Introduction

Intraocular pressure (IOP) change in fellow untreated eyes accompanied by a corresponding IOP reduction in the operated eye has been identified as the “consensual ophthalmotonic reaction” (COR) and was firstly described in 1924 by Weekers through an experimental study [1]. This response was shown after contusions, ocular compression, tonography, and cauterization of the sclera [2–4]. Later COR was described in glaucoma patients following unilateral use of topical IOP-lowering medications [5–11]. A retrospective review by Gibbens indicated a fellow eye IOP response after monocular application of antiglaucomatous drops in 13 patients with ocular hypertension [12]. The Ocular Hypertension Treatment Study showed IOP reduction in the untreated fellow eye of approximately 1.5 mmHg after initiation of unilateral topical beta-blocker therapy in over 700 patients [5]. This effect could be explained as systemic absorption and transfer of the topical beta-blocker to the fellow eye via the bloodstream, as was proposed by Zimmerman and Kaufman [6]. In contrast to Gibbens, Newman et al. reported an IOP reduction in fellow untreated eyes of 38 patients with ocular hypertension or glaucoma only with beta-blocker and not with other topical IOP-lowering medications [13]. IOP reduction of 8–11.2% from baseline in the fellow eye of glaucoma patients was reported after laser therapy [14–16].

The first report on COR after a fistulating surgery in experimental animals was published by Wilmer in 1927 [17]. Similar IOP reduction in the fellow eye was shown in glaucoma patients after penetrating surgery [18–20]. The first report on IOP elevation in the fellow eye after unilateral paracentesis was published by Al-Ghadyan et al. investigating rabbits [21]. The incidence for IOP rise in the fellow eye following surgery in humans has been reported by Simmons to be around 10% [22]. Several other studies have shown the possibility of IOP elevation in the fellow eye after unilateral glaucoma surgery including not only trabeculectomy (TE) but glaucoma drainage device (ExPress shunt®, Ahmed Valve, Aurolab Aqueous Drainage Device) implantations as well [23–25]. Thus, there is still a controversial discussion in the literature whether there is an IOP decrease, increase, or no change in the fellow eye after IOP-lowering surgery.

In addition, glaucoma surgery has undergone changes in recent years. Less invasive techniques using small stents for subconjunctival filtration have been developed recently. We therefore analyzed the effect of PreserFlo™ microshunt implantation (PMI) in this study as well. Filtering canaloplasty (FCP) is a technique that has been recently developed [26]. We wanted to know whether these three techniques cause inter-eye changes of IOP after surgery to shed light on systemic effects of unilateral surgery.

## Materials and methods

We performed a retrospective clinical study including 235 patients who underwent mitomycin C (MMC)–augmented TE, FCP, or PMI as initial unilateral glaucoma surgery from January 2019 to January 2020 at the Department of Ophthalmology of the University Medical Center of the Johannes Gutenberg-University Mainz, Germany. Initial TE was performed in 187 patients, FCP in 25 patients, and PMI in 23 patients in one (treated) eye, while the fellow eye had not had any previous glaucoma surgery. All surgeries were performed by two experienced glaucoma surgeons (EMH, PC). The study adhered to the tenets of the Declaration of Helsinki and was approved by the ethics committee of the medical board of Rhineland-Palatinate, Germany.

Indication for surgery was deterioration of optic nerve head, retinal nerve fiber layer thickness, or of visual field. Decision was made on a clinical basis, and informed consent was obtained in all patients.

For this study, main outcome measure was IOP change in the fellow eye. Secondary outcomes were proportion of clinically significant IOP elevation (occurrence of postoperative IOP > 21 mmHg with preoperative IOP ≤ 21 mmHg) in the fellow eye during follow-up and evaluation of potential risk factors (predictive factors) associated with IOP change. The amount and percentage of IOP change from preoperative level in the fellow eye was evaluated for each postoperative follow-up.

Patients who underwent simultaneous bilateral glaucoma surgeries or had only one functional eye (monocular patients) were excluded. Two patients who stopped topical therapy in the fellow eye before surgery were also excluded from further analysis. According to the preoperative standard operating procedure of the Mainz University Eye Hospital, patients discontinued topical medication in the eye scheduled for surgery 2–4 weeks preoperatively and treated with systemic acetazolamide during this time to reduce inflammation in the conjunctiva, except 4 patients with intolerance to acetazolamide. Five days preoperatively, preservative-free topical steroids (Dexa EDO 1.3 mg/mL) were administered 4 times daily. Systemic acetazolamide was discontinued postoperatively in all patients.

Preoperative IOP was taken at the time of surgery indication and was defined as the median of three IOP measurements in every patient. In most of our patients, day-and-night IOP profiles were performed by local ophthalmologists or in our clinic. Postoperatively, IOP was measured on day 1, day 2, and at 1 week after surgery. We took only morning IOP measurements.

### Surgery

#### Trabeculectomy

Local, general, or combined anesthesia was applied. A fornix-based conjunctival peritomy (6 mm) at the limbus was performed. MMC with concentration 0.2 mg/mL and exposure time 3 min was applied subconjunctivally, followed by irrigation with 30 mL of balanced salt solution. A partial-thickness scleral flap of size 4 × 4 mm was created. A paracentesis wound was created with a 15 degree side port knife at the temporal clear cornea. During TE, Schlemm’s canal (SC) and trabecular meshwork (TM) excision was made with a 15 degree stab knife and a surgical basal peripheral iridectomy was performed.

#### Filtering canaloplasty

This method, developed at our department, is a modification of common suture canaloplasty. It provides a three-way outflow for the aqueous humor: first, by SC dilation and suturing; second, by deep second scleral flap dissection for suprachoroidal outflow enhancement; and third, by allowing some filtration over time, supported by the application of MMC.

The second deep scleral flap of size 1.5 × 3 mm was created within the borders of the superficial flap dissecting forward, exposing the choroid, into the clear cornea. The roof of SC was carefully detached with multifunctional 25-gauge forceps. The inner scleral flap was removed. Then, insertion of iTrack™ catheter (iTrack Surgical System Ellex, Distributor Ruck, Germany) was performed over 360 degree. Every 2 clock hours, a precise amount of Healon® PRO was injected into SC via the screw-driven injector. The optical fiber that illuminated the tip of the microcatheter provided guidance to the path of the catheter as it was advanced. Care was taken to keep the catheter in perpetual motion through SC when viscoelastic was injected to prevent the creation of a Descemet membrane detachment. When the distal tip of the catheter re-emerged from the opposite opening of SC, a 10–0 polypropylene (Prolene) suture was tied to it and the microcatheter was withdrawn from the canal. Prolene suture was detached from the catheter and tightened to apply a moderate tension to the tissues of the inner wall of SC to the extent of visible slight indentation of the outer SC.

The scleral flap in FCP as well as in TE was then closed loosely with up to 10.0 Nylon single-stitch sutures (at discretion of the surgeon) to allow gentle subconjunctival filtration.

#### PreserFlo™ microshunt

After conjunctival opening at the limbus and subconjunctival preparation, wet field cautery of episcleral veins was performed. MMC was applied on three sponges (3 min) under the tenon and washed out with 30 mL BSS. After marking the sclera 3 mm from the limbus, a 1-mm-deep scleral pocket was made using a triangular knife. A 25-gauge needle was introduced into the pocket and a scleral needle track was created into the anterior chamber followed by insertion of the microshunt through this track into the anterior chamber. The fins of the shunt were anchored in the scleral pocket. After checking for flow, tenon is adapted anteriorly with two 10.0 Nylon single sutures followed by an interlocked watertight running 10.0 Nylon suture in a meander-like fashion (Mainz suture) used in all 3 types of filtering surgery [27].

Subconjunctival injection of 4 mg dexamethasone was performed in the inferior fornix of the conjunctiva, and then, ofloxacin ointment was applied prior bandage.

Postoperative topical medications included dexamethasone 0.1% drops 6 times a day (tapering off over 6 weeks), 0.3% ofloxacin 4 times a day (5 days), and 1% prednisolone pivalate ointment once a day (2 weeks). In cases of transient hypotony with anterior chamber shallowing, atropine 1% was prescribed.

### Statistical analysis

Absolute and relative frequencies were computed for dichotomous data; continuous data are presented as mean ± standard deviation and median respective inter-quartile range. Paired Wilcoxon test was used to compare preoperative and postoperative IOP in both eyes. Correlations between the IOP change from baseline to follow-up in the treated eye and the IOP change in the fellow eye were analyzed using Spearman’s rank correlation. Predictive factors for IOP change in the fellow eye were analyzed using linear regression analysis with gender, age, preoperative IOP in the fellow eye, previous cataract extraction, IOP change in the operated eye, and duration of glaucoma as independent variables. All statistical analyses were conducted with R (R Core Team 2020, R Foundation for Statistical Computing, Vienna, Austria).

## Results

Demographic and clinical data, including type of glaucoma and surgical status, are shown in Table 1.

**Table 1:** Demographic and clinical data of all patients

Data	Type of performed glaucoma surgery		
	TE	FCP	PMI
Number of patients	187	25	23
Glaucoma diagnosis			
Unilateral/bilateral	6 (3.2%)/181 (96.8%)	-/25 (100%)	1 (4.3%)/22 (95.7%)
Operated eye			
Right/left	107 (57.2%)/80 (42.8%)	10 (40%)/15 (60%)	8 (34.8%)/15 (65.2%)
Mean age (range), years	67.1 ± 10.4 (40–88)	60.2 ± 12.6 (35–84)	68.6 ± 13.3 (25–86)
Sex, female/male	96 (51%)/91 (49%)	10 (40%)/15 (60%)	14 (61%)/9 (39%)
Type of glaucoma			
POAG	111 (59%)	18 (72%)	10 (44)
PEX glaucoma	33 (18%)	3 (12%)	5 (22%)
NTG	23 (12%)	2 (8%)	6 (26%)
Pigmentary glaucoma	9 (5%)	2 (8%)	–
Secondary uveal glaucoma	7 (4%)	–	2 (9%)
Other	4 (2%)	–	–
Mean duration of glaucoma, years	8.1 ± 8.5	9.4 ± 6.5	7.6 ± 6.6
Presence of topical therapy in the fellow eye	170 (91%)	24 (96%)	19 (83%)
Surgical status of eye			
Previous laser trabeculoplasty			
Treated eye/fellow eye	22 (12%)/18 (10%)	3 (12%)/3 (12%)	1 (4%)/1 (4%)
Previous cataract surgery			
Treated eye/fellow eye	75 (40%)/62 (33%)	7 (28%)/6 (24%)	16 (70%)/16 (70%)
Previous vitreoretinal surgery			
Treated eye/fellow eye	7 (4%)/4 (2%)	1 (4%)/-	-/-
*T*_max_ (mmHg), treated eye/fellow eye	31.1 ± 9/30.6 ± 9	31.8 ± 11.6/32.2 ± 13.1	31.6 ± 10.4/29.7 ± 8.9
CCT (μm), treated eye/fellow eye	524.6 ± 37.3/526.2 ± 34.3	517.7 ± 29.6/516.6 ± 34.2	527.3 ± 25.5/532.3 ± 25.2
CDVA, treated eye/fellow eye	0.7 ± 0.3/0.8 ± 0.3	0.7 ± 0.3/0.8 ± 0.2	0.7 ± 0.3/0.7 ± 0.3
MD (dB), treated eye/fellow eye	12.4 ± 6.9/6.9 ± 5.5	11.7 ± 6.8/7.7 ± 6.7	10.6 ± 5.7/7.2 ± 6.8

*TE*, trabeculectomy; *FCP*, filtering canaloplasty; *PMI*, PreserFlo^TM^ microshunt implantation; *SD*, standard deviation; *POAG*, primary open angle glaucoma; *PEX glaucoma*, pseudoexfoliative glaucoma; *NTG*, normal tension glaucoma; *T*_*max*_, maximum intraocular pressure; *CCT*, central corneal thickness; *CDVA*, corrected distance visual acuity; *MD*, mean deviation (Octopus perimetry)

### Trabeculectomy group

**Fig. 1:**
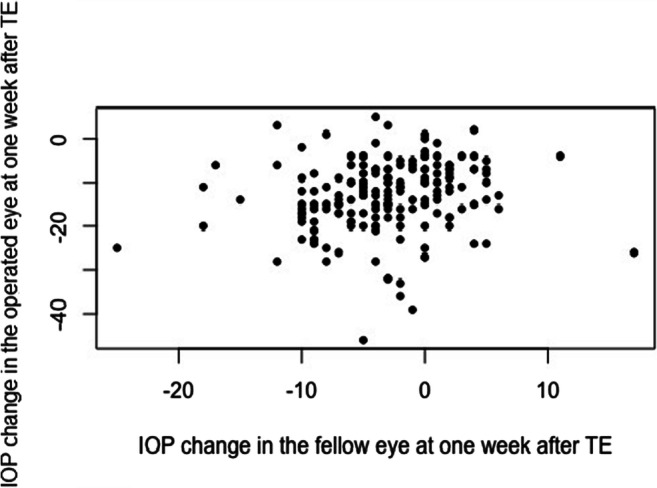
Correlation between IOP changes from baseline in the operated and in the fellow eye of patients at 1 week after trabeculectomy (rho = 0.24, *p* = 0.001). TE, trabeculectomy

The mean age of patients in the TE study group was 67.1 ± 10.4 years. The median (range) preoperative and postoperative IOP in the treated eye on the 1st and 2nd days and at 1 week after TE were 20 (17–26) mmHg, 13 (9–20) mmHg, 12 (8–18.3) mmHg, and 9 (5–11) mmHg, respectively. The median preoperative and postoperative IOP in the fellow eye on the 1st and 2nd days and at 1 week after TE were 17 (14–20) mmHg, 16 (14–21) mmHg, 15 (13–18) mmHg, and 14 (12–17) mmHg, respectively. In 148 (79.1%) fellow eyes, preoperative IOP was ≤ 21 mmHg, and in 7 (3.7%) fellow eyes, preoperative IOP was ≥ 30 mmHg. There was no statistically significant difference between preoperative IOP and IOP in the fellow eye on the 1st day after surgery (*p* = 0.10), but IOP at 1 week after TE was statistically significantly lower than that preoperatively (*p* < 0.0001). The median IOP change in the operated eyes was − 8 (− 14 to 0) mmHg and in the fellow eyes 0 (− 5 to 3) mmHg on the 1st day after TE. At 1 week after TE, the median IOP change in the treated eyes was − 12 (− 18 to − 7) mmHg and in the fellow eyes − 3 (− 6 to 0) mmHg. The higher the IOP reduction was in the surgical eye, the larger was the IOP reduction in the fellow eye (rho = 0.24, *p* = 0.001) (Fig. 1). IOP elevation occurred in 33% and 22% of fellow eyes on the 1st postoperative day and at 1 week after TE, respectively (Table 2).

**Table 2:** Percentage of IOP change from baseline in the fellow eye on the 1st day and at 1 week after surgery

Percentage of IOP change	TE, % of fellow eyes		FCP, % of fellow eyes		PMI, % of fellow eyes	
	1st day	1 week	1st day	1 week	1st day	1 week
IOP reduction						
≥ 30%	13%	42%	4%	12%	9%	13%
≥ 50%	2%	4%	4%	–	4%	4%
IOP elevation	33%	22%	16%	32%	35%	35%
≥ 30%	9%	8%	8%	4%	13%	9%
≥ 50%	5%	3%	8%	–	9%	4%

*TE*, trabeculectomy; *FCP*, filtering canaloplasty; *PMI*, PreserFlo^TM^ microshunt implantation

### Filtering canaloplasty group

The mean age of patients in the FCP study group was 60.2 ± 12.6 years. The median (range) preoperative and postoperative IOP in the treated eye on the 1st and 2nd days and at 1 week after FCP were 18 (16–21) mmHg, 10 (7.5–12) mmHg, 10 (8–12) mmHg, and 11.5 (8–12) mmHg, respectively. The median preoperative and postoperative IOP in the fellow eye on the 1st and 2nd days and at 1 week after FCP were 17 (16–19) mmHg, 17 (14.5–18.5) mmHg, 16.5 (13.5–18.3) mmHg, and 17 (14–18.5) mmHg, respectively. In 21 (84%) fellow eyes, preoperative IOP was ≤ 21 mmHg; in 2 (8%) fellow eyes, preoperative IOP was ≥ 30 mmHg. There was not any statistically significant difference between preoperative IOP and IOP in the fellow eye on the 1st day and at 1 week after surgery (*p* = 0.33 and *p* = 0.10). The median IOP change in treated eyes was − 10 (− 14 to − 4) mmHg and − 2 (− 3 to 0.5) mmHg in fellow eyes on the 1st day after FCP. The median IOP change in the operated eyes at 1 week after FCP was − 7.5 (− 11.3 to − 5) mmHg and − 2 (−4 to 1.5) mmHg in fellow eyes. No correlation was found between IOP changes between both eyes. IOP elevation occurred in 16% and 32% of fellow eyes on the 1st postoperative day and at 1 week after FCP, respectively.

### PreserFlo™ microshunt group

**Fig. 2:**
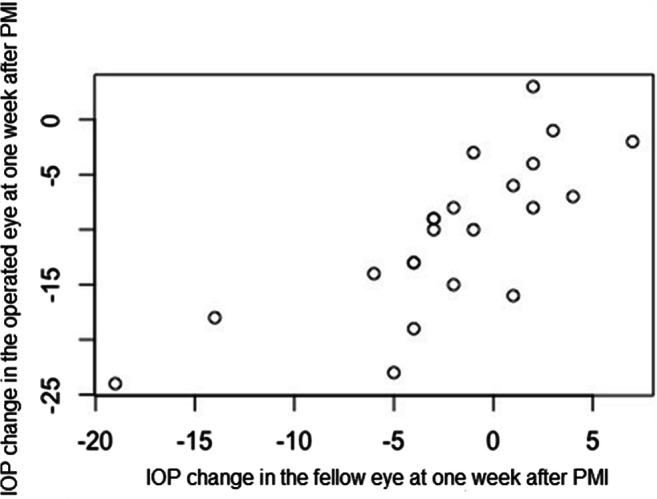
Correlation between IOP changes from baseline in the operated and in the fellow eye of patients at 1 week after PreserFlo™ microshunt implantation (rho = 0.82, *p* < 0.0001). PMI, PreserFlo™ microshunt implantation

The mean age of patients in the PMI group was 68.6 ± 13.3 years. The median preoperative and postoperative IOP in the treated eye on the 1st and 2nd days and at 1 week after PMI were 17 (14.5–20.5) mmHg, 7 (5–9.5) mmHg, 7 (5–8) mmHg, and 7 (6–8.8) mmHg, respectively. The median preoperative and postoperative IOP on the 1st and 2nd days and at 1 week after PMI in the fellow eye were 15 (12.5–18) mmHg, 14 (13–16) mmHg, 16 (12.5–18) mmHg, and 14 (12–16) mmHg, respectively. In 20 (87%) fellow eyes, preoperative IOP was ≤ 21 mmHg; in 1 (4%) fellow eye, preoperative IOP was ≥ 30 mmHg. There was not any statistically significant difference between preoperative IOP and IOP in the fellow eye on the 1st day and at 1 week after surgery (*p* = 0.67 and *p* = 0.08). The median IOP change in treated eyes was − 10 (− 14.5 to − 7) mmHg and − 0.5 (− 3 to 2.8) mmHg in fellow eyes on the 1st day after PMI. The median IOP change in treated eyes at 1 week after PMI was − 9.5 (− 14.8 to − 6.3) mmHg and − 2 (− 4 to 1.8) mmHg in fellow eyes, with significant correlation between both eyes (rho = 0.82; *p* < 0.0001) (Fig. 2). IOP elevation was found in 35% of fellow eyes both on the 1st day and at 1 week after PMI; in 9% of fellow eyes, this IOP rose more than 50% from baseline on the 1st day after PMI.

### All groups

The median preoperative and postoperative IOP in the treated eye on the 1st and 2nd days and at 1 week after surgery were 20 (17–26) mmHg, 11 (8–18) mmHg, 11 (7–18) mmHg, and 9 (6–12) mmHg, respectively. The median preoperative and postoperative IOP on the 1st and 2nd days and at 1 week after surgery in the fellow eye were 17 (14–20) mmHg, 16 (14–19) mmHg, 16 (13–18) mmHg, and 14 (12–17) mmHg, respectively. There was not a statistically significant difference between preoperative IOP and IOP in the fellow eye on the 1st day after surgery (*p* = 0.06), but IOP at 1 week after filtering surgery was statistically significantly lower than that preoperatively (*p* < 0.0001). The median IOP change in treated eyes was − 8 (− 14 to − 2) mmHg and − 0.5 (− 4 to 3) mmHg in fellow eyes on the 1st day after surgery. The median IOP change in treated eyes at 1 week after surgery was − 11 (− 17 to − 7) mmHg and − 3 (− 5.5 to 1) mmHg in fellow eyes, with significant correlation between both eyes (rho = 0.33; *p* < 0.0001).

### Intraocular pressure elevation in fellow eyes after surgery

IOP elevation (postoperative IOP > 21 mmHg) within 1 week follow-up period after surgery in fellow eyes with preoperative IOP ≤ 21 mmHg (189 eyes) was detected in 24 (12.7%) eyes. The cumulative proportion of unexplained IOP elevation was 14.2% (21 from 148 eyes) after TE, 9.5% (2 from 21 eyes) after FCP, and 5% (1 from 20 eyes) after PMI. All IOP-lowering medications were continued in the fellow eye of 22 patients. Two patients who were not on fellow eye medication before surgery were initiated on therapy during hospital stay due to elevated IOP. In 75% fellow eyes, IOP elevation was discovered on the 1st postoperative day, in 41.7% eyes on the 2nd postoperative day, and in 20.8% eyes at 1 week after surgery. In 7 (33.3%) TE patients, IOP rise in the fellow eye occurred at 2 or more follow-ups. Nine (37.5%) patients demonstrated fellow eye IOP rise > 50% from baseline. 11.1% and 20% of the patients with IOP > 21 mmHg in the fellow eye on the 1st and 2nd postoperative days, respectively, and no patient with IOP > 21 mmHg in the fellow eye at 1 week after surgery had transient hypotony in the treated eye, associated with overdraining bleb without requirement for further surgical intervention.

**Fig. 3:**
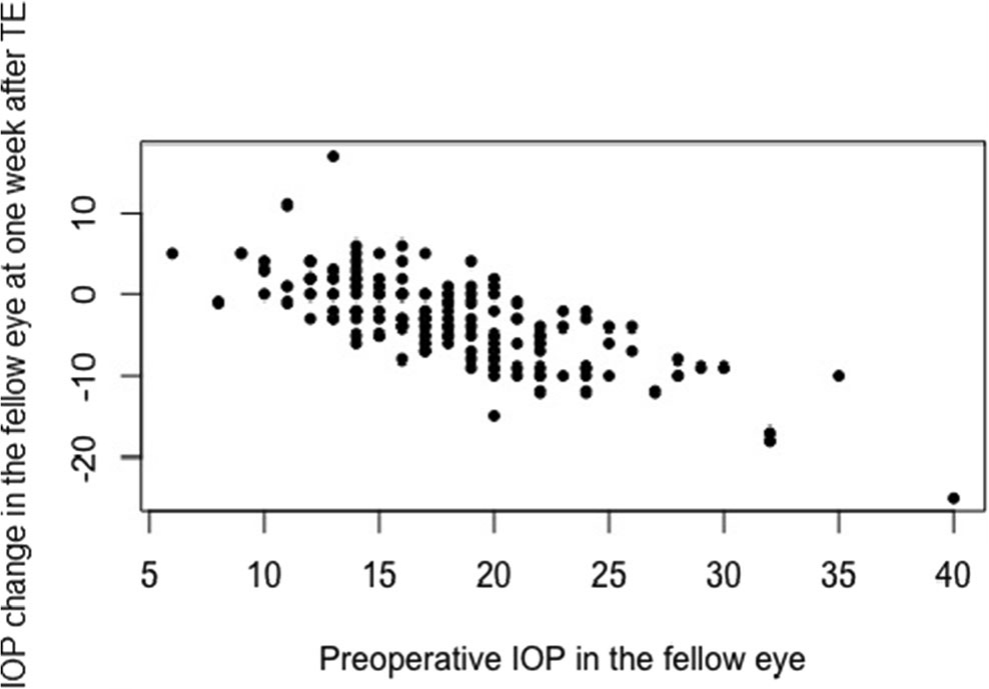
Correlation between preoperative IOP in the fellow eye and IOP change from baseline at 1 week after trabeculectomy (*p* < 0.0001). TE, trabeculectomy

**Fig. 4:**
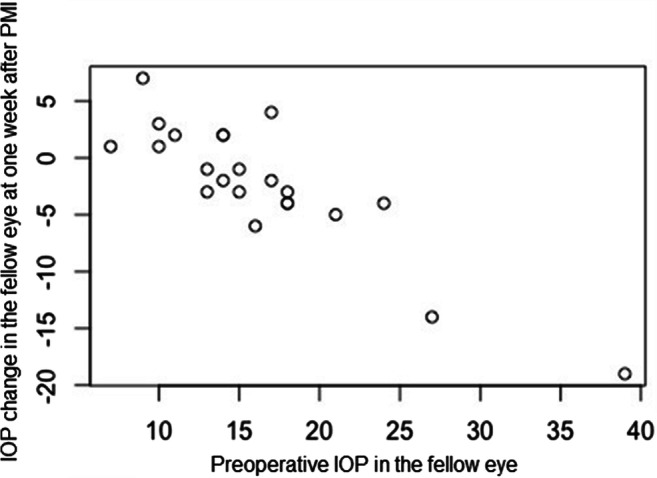
Correlation between preoperative IOP in the fellow eye and IOP change from baseline at 1 week after PreserFlo™ microshunt implantation (*p* < 0.01). *PMI*, PreserFlo™ microshunt implantation

Linear regression analysis revealed preoperative IOP in fellow eye as a statistically associated factor for IOP change in the fellow eye after 1 week, while age, gender, phakic status of the fellow eye, IOP change in the treated eye at 1 week after surgery, and duration of glaucoma were not associated (Table 3). Consequently, the higher the preoperative IOP was in fellow eyes, the larger was the IOP-lowering effect at 1 week after TE (Fig. 3) and after PMI (Fig. 4).

**Table 3:** Factors associated with IOP change in the fellow eye at 1 week after trabeculectomy and PreserFlo™ microshunt implantation and all types of filtering surgery

**Factor**	**TE**			**PMI**		
	**Estimate**	**Standard error**	***p*** **value**	**Estimate**	**Standard error**	***p*** **value**
Gender	− 1.02	0.55	0.06	− 1.37	1.53	0.39
Age	− 0.03	0.03	0.35	0.004	0.07	0.95
Preoperative IOP in the fellow eye	− 0.79	0.06	< 0.0001^*^	− 0.60	0.15	< 0.01^*^
Previous cataract extraction in the fellow eye	− 1.02	0.65	0.11	− 2.42	2.36	0.32
IOP change in the operated eye at 1 week	− 0.05	0.04	0.17	0.16	0.16	0.32
Duration of glaucoma	0.04	0.03	0.18	0.03	0.12	0.81
**Factor**	**All types of filtering surgery**					
	**Estimate**	**Standard error**	***p*** **value**			
Gender	− 0.89	0.47	0.06			
Age	− 0.04	0.02	0.14			
Preoperative IOP in the fellow eye	− 0.76	0.04	< 0.0001^*^			
Previous cataract extraction in the fellow eye	− 1.1	0.56	< 0.05^*^			
IOP change in the operated eye at 1 week	− 0.03	0.03	0.31			
Duration of glaucoma	0.05	0.03	0.1			

*TE*, trabeculectomy; *PMI*, PreserFlo™ microshunt implantation. ^*^p < .05

Twenty-two (9.4%) patients had not received any IOP-lowering drops in the fellow eye because of unilateral glaucoma (31.8%) or intolerance to antiglaucomatous drops (68.2%). Subgroup analysis was performed in TE patients with fellow eyes without topical therapy and fellow eyes received topical IOP-lowering drops, respectively. Fellow eyes on topical therapy showed a statistically significant difference between preoperative and postoperative IOP on the1st postoperative day and at 1 week following surgery, while fellow eyes without topical therapy showed no significant difference (*p* = 0.02 vs *p* = 0.72 and *p* = 0.0001 vs *p* = 0.08) (see Table, Supplemental Digital Content 1, which demonstrates subgroup analysis).

## Discussion

This study investigates the IOP response in the fellow eye of patients following initial TE, FCP, or PMI in the early postoperative period. We noted a statistically significant decrease in IOP in the fellow eyes of TE patients at 1 week after surgery.

Published results on the topic of fellow eye IOP response after unilateral IOP-lowering surgery are controversial, due to differences in the design of performed studies, inclusion criteria, surgical technique, and length of follow-up. The largest prospective evaluation to date of untreated fellow eye response included 300 glaucoma patients after unilateral TE within the framework of the Collaborative Initial Glaucoma Treatment Study (CIGTS). Radcliffe et al. did not find any evidence of a substantial effect of TE on the fellow eye IOP during follow-up. However, this study is limited by the absence of IOP data concerning the fellow eye in the early postoperative period, and by a significant number of censoring events, such as glaucoma or cataract surgery, or laser treatment in the fellow eyes [18]. Vysniauskiene et al. reported an IOP reduction in fellow eyes of 24 patients in the early postoperative period after TE, but half of their patients had undergone previous TE in the fellow eye, and among those patients COR was less pronounced [20].

Shum et al. described a mean IOP rise in the fellow eyes of 22 Chinese glaucoma patients, following augmented TE or ExPress shunt® implantation during the 2 weeks following surgery. Twenty-three percent of patients demonstrated fellow eye IOP rise > 30% from baseline. Small sample size, surgical results of different surgeons, and performance of IOP measurement by different tonometers are the most significant limitations of this study [24]. Yarangumeli et al. reported an IOP rise in 33% of fellow eyes. A consistent IOP elevation of 50% or more was found in 8% of fellow eyes during the first 3 months after TE [23]. Kaushik showed a maximum IOP rise in the fellow eye at 6 weeks following TE, Ahmed valve, and Aurolab Aqueous Drainage Device implantations [25]. A leaking or overdraining bleb with the requirement for surgical intervention was observed in the treated eyes of most of the patients with a fellow eye IOP rise of greater than 50% from baseline [18, 24]. In our study, an IOP rise on the 1st postoperative day and a clinically significant IOP rise in fellow eyes within the follow-up period were identified in 31% and in 12.7% of all included patients, respectively. In contrast, no patient with a clinically significant IOP rise and a postoperative IOP of greater than 21 mmHg in the fellow eye required further surgical intervention in the treated eye.

We found a correlation between IOP changes from baseline in the treated and in the fellow eyes at 1 week after surgery in the TE and PMI groups, as previously reported after TE [18, 23, 24]. In the CIGTS-associated study, a higher baseline IOP, a lower level of education, and time point of observation were evaluated as associated factors for higher fellow eye IOP [18]. In our study, a higher baseline IOP in the fellow eye of TE and PMI patients was a predictive factor for a larger postoperative IOP-lowering effect.

IOP reduction in the fellow eye could be associated with improved medication compliance of patients as in-patients, the continuation of acetazolamide effect taken preoperatively, or the presence of a central nervous system (CNS)–mediated contralateral effect. As TE is usually planned when the IOP is high on the variation curve, the IOP reduction in the fellow eye could also be the result of a “regression to the mean” effect [25, 28]. According to the published data, there are significant postoperative IOP changes in the fellow eye after unilateral TE in the treated eye, irrespective of whether the fellow eye was on topical antiglaucomatous medications or not [20, 23, 25]. Our subgroup analysis revealed that fellow eyes with no IOP-lowering drops showed no difference between preoperative and postoperative IOP, in contrast to fellow eyes on topical therapy. These findings might provide support for the argument that IOP reduction in the fellow eye is probably due to improved postoperative compliance, especially because our patients were in-patients during most of the postoperative period. In terms of central regulation of IOP, first postulation was done by Leplat, who found that controlled blunt trauma to a rabbit eye produced a more marked COR if the animal was not anesthetized [29]. Several experimental studies have shown that stimulation of the hypothalamus may be followed by an alteration of IOP [30–32]. Prijot and Stone suggested a possible role of the parasympathetic nervous system [2] and Gibbens demonstrated the role of the sympathetic nervous system in limbs of the COR [33]. The CNS-mediated reflex could also be responsible for the IOP elevation in the fellow eye after unilateral glaucoma surgery. Other possible explanations for the IOP rise in the fellow eye include the postoperative withdrawal of IOP-lowering medications or the postoperative withdrawal of acetazolamide. Kaushik et al. showed that there was no significant difference in the mean IOP rise with and without preoperative acetazolamide [25]. An increase in flow rate from 2.56 to 2.90 mL/min after TE was identified by computerized fluorophotometry, used by Diestelhorst and Krieglstein. This rise might cause IOP elevation in a predisposed eye with relatively poor outflow capacity [34]. Glaucoma surgery leads to underperfusion of the remaining TM by aqueous humor in the treated eye, followed by deposition of increased extracellular TM material within the cribriform region. A reflex mechanism, mediated by specialized cells in the scleral spur, leads to decreased outflow also in the fellow eye [23, 35–37].

One of the strengths of our study is that we had included only subjects with fellow eyes without any previous glaucoma surgery. Measurement time of the IOP was identical to reduce the effect of daily fluctuation of IOP. Limitations are its retrospective design and the administration of systemic acetazolamide preoperatively to 98% of the included patients. Additionally, 91% of the included patients received IOP-lowering medications in both eyes before surgery that was planned and indicated by the glaucoma surgeon. Thus, it was impossible to analyze the influence, if any, of different medication regimens on IOP changes in the fellow eyes of these patients.

To our knowledge, this report is unique in that it is the first report to evaluate the fellow eye IOP response in the early postoperative period and to analyze different types of initial unilateral glaucoma surgery. Our study shows a statistically significant IOP-lowering effect in the un-operated fellow eye at 1 week only after TE, and no significant effect due to FCP or PMI on fellow eye IOP during the early postoperative period. Despite the fact that TE does not appear to increase the mean IOP of the fellow eye—as suggested by several earlier studies on this topic—an IOP rise is identified in almost one-third of fellow eyes in all our study groups, and a significant IOP rise might occur in the fellow eye of some glaucoma patients after all mentioned types of glaucoma surgery.

Significant IOP elevation in the fellow eyes of patients with advanced glaucoma and already on maximal medical therapy could have a clinical impact and influence further clinical management. Therefore, attention should be paid to follow-up processes after unilateral filtering surgery. It is recommended to inform each patient preoperatively about the possibility of IOP change in the fellow eye after penetrating surgery, and to postoperatively measure IOP in both eyes, especially in the early postoperative period.

## Supplementary information

ESM 1 (PDF 13 kb)

## Data Availability

The authors confirm that the data supporting the findings of this study are available within the article and its supplementary materials. The partial data that support the findings of this study are openly available in the journal “Der Ophthalmologe” 2020·117 (Suppl 2, DOG 2020 Abstracts):S43–S190 at 10.1007/s00347-020-01197-0.

## References

[1] Weekers L (1924). Modification experimentales de l’ophtalmotonous. Reaction ophtalmotonique consenuelle. Arch Ophthalmol (Paris).

[2] Prijot EL, Stone HH (1956). On the ophthalmotonic consensual reaction and its relationship to aqueous humor dynamics. Am J Ophthalmol.

[3] Nagata N, Kurimoto S, Matsuka M (1954). A study of consensual ophthalmotonic reaction. Acta Soc Ophthalmol Japan.

[4] Stocker FW (1958). On changes in intraocular pressure after application of the tonometer. Am J Ophthalmol.

[5] Piltz J, Gross R, Shin DH, Beiser JA, Dorr DA, Kass MA, Gordon MO (2000). Ocular hypertension treatment study group. Contralateral effect of topical beta-adrenergic antagonists in initial one-eyed trials in the ocular hypertension treatment study. Am J Ophthalmol.

[6] Zimmerman TJ, Kaufman HE (1977). Timolol: a beta-adrenergic blocking agent for the treatment of glaucoma. Arch Ophthalmol.

[7] Kwitko GM, Shin DH, Ahn BH, Hong YJ (1987). Bilateral effects of long-term monocular timolol therapy. Am J Ophthalmol.

[8] Drance SM (1980). The uniocular therapeutic trial in the management of elevated intraocular pressure. Surv Ophthalmol.

[9] Dunham CN, Spaide RF, Dunham G (1994). The contralateral reduction of intraocular pressure by timolol. Br J Ophthalmol.

[10] Rao HL, Senthil S, Garudadri CS (2014). Contralateral intraocular pressure lowering effect of prostaglandin analogues. Indian J Ophthalmol.

[11] King AJ, Rotchford AP (2016). Validity of the monocular trial of intraocular pressure-lowering at different time points in patients starting topical glaucoma medication. JAMA Ophthalmol.

[12] Gibbens MV (1988). The consensual ophthalmotonic reaction. Br J Ophthalmol.

[13] Newman H, Kurtz S, David R (2010). Intraocular pressure changes in the contralateral eye after topical treatment: does an “ophthalmotonic consensual reaction” exist?. Isr Med Assoc J.

[14] Rhodes KM, Weinstein R, Saltzmann RM, Aggarwal N, Kooner KS, Petroll WM, Whitson JT (2009). Intraocular pressure reduction in the untreated fellow eye after selective laser trabeculoplasty. Curr Med Res Opin.

[15] Latina MA, Sibayan SA, Shin DH, Noecker RJ, Marcellino G (1998). Q-switched 532-nm Nd:YAG laser trabeculoplasty (selective laser trabeculoplasty): a multicenter, pilot, clinical study. Ophthalmology.

[16] McIlraith I, Strasfeld M, Colev G, Hutnik CM (2006). Selective laser trabeculoplasty as initial and adjunctive treatment for open-angle glaucoma. J Glaucoma.

[17] Wilmer WE (1927). Discussion on the results of operative treatment of glaucoma. Trans Ophthalmol Soc UK.

[18] Radcliffe NM, Musch DC, Niziol LM, Liebmann JM, Ritch R (2010). Collaborative Initial Glaucoma Treatment Study Group. The effect of trabeculectomy on intraocular pressure of the untreated fellow eye in the collaborative initial glaucoma treatment study. Ophthalmology.

[19] Detorakis ET, Tsiklis N, Pallikaris IG, Tsilimbaris MK (2011). Changes in the intraocular pressure of fellow untreated eyes following uncomplicated trabeculectomy. Ophthalmic Surg Lasers Imaging.

[20] Vysniauskiene I, Shaarawy T, Flammer J, Haefliger IO (2005). Intraocular pressure changes in the contralateral eye after trabeculectomy with mitomycin C. Br J Ophthalmol.

[21] Al-Ghadyan A, Mead A, Sears M (1979). Increased pressure after paracentesis of the rabbit eye is completely accounted for by prostaglandin synthesis and release plus pupillary block. Invest Ophthalmol Vis Sci.

[22] Simmons RJ, Chandler PA, Grant WM, Epstein DL (1986). Filtering operations. Chandler and Grant’s glaucoma.

[23] Yarangumeli A, Koz OG, Kural G (2003). The effect of trabeculectomy on the intraocular pressure of the unoperated fellow eye. J Glaucoma.

[24] Shum JW, Choy BN, Ho WL, Chan JC, Lai JS (2016). Consensual ophthalmotonic reaction in Chinese patients following augmented trabeculectomy or ExPRESS shunt implantation. Medicine (Baltimore).

[25] Kaushik S, Agarwal A, Kaur S, Lomi N, Raj S, Pandav SS (2016). Change in intraocular pressure in the fellow eye after glaucoma surgery in 1 eye. J Glaucoma.

[26] Liebezeit S, Prokosch-Willing V, Grehn F (2017). Methodik der Mainzer modifi zierten Kanaloplastik – neue Behandlungsstrategie in der Glaukomchirurgie. Der Ophthalmologe: Zeitschrift der Deutschen Ophthalmologischen Gesellschaft.

[27] Pfeiffer N, Grehn F (1992). Improved suture for fornix-based conjunctival flap in filtering surgery. Int Ophthalmol.

[28] Leung DY, Kwong YY, Yuen HK (2006). Intraocular pressure changes in the contralateral eye after trabeculectomy with mitomycin C [letter]. Br J Ophthalmol.

[29] Leplat G (1924). Etude de quelques reactions dans les yeux par une contusion oculaire unilateral; recherches experimentales et cliniques. Ann Oculist (Paris).

[30] Schmerl E, Steinberg B (1948). Central control of intraocular pressure by active principles. Am J Ophthalmol.

[31] Gloster J, Greaves DP (1957). Effect of diencephalic stimulation upon intraocular pressure. Br J Ophthalmol.

[32] Cox CE, Fitzgerald CR, King RL (1975). A preliminary report on the supraoptic nucleus and control of intraocular pressure. Investig Ophthalmol.

[33] Gibbens MV (1988). Sympathetic influences on the consensual ophthalmotonic reaction. Br J Ophthalmol.

[34] Diestelhorst M, Krieglstein G (1991). The effect of trabeculectomy on the aqueous humor flow of the unoperated fellow eye. Graefes Arch Clin Exp Ophthalmol.

[35] Lutjen-Drecoll E, Barany EH (1974). Functional and electron microscopic changes in the TM remaining after trabeculectomy in cynomolgus monkeys. Investig Ophthalmol.

[36] Kaufman PL, Ritch R, Shields MB, Krupin T (1996). Pressure dependent outflow. The glaucomas.

[37] Tamm ER, Flugel C, Stefani FH, Lütjen-Drecoll E (1994). Nerve endings with structural characteristics of mechanoreceptors in the human scleral spur. Invest Ophthalmol Vis Sci.

