# Supplementary material for: Inter-eye relationship of intraocular pressure change after unilateral trabeculectomy, filtering canaloplasty, or PreserFlo™ microshunt implantation
Source: Graefes Arch Clin Exp Ophthalmol. 2021 May 8;259(10):3045–53. doi: 10.1007/s00417-021-05188-y (PMC8478758; doi:10.1007/s00417-021-05188-y)
Supplement: Supplementary file 1 — (PDF 13 kb) [file 417_2021_5188_MOESM1_ESM.pdf]

Supplemental Digital Content 1. Subgroup analysis in patients underwent trabeculectomy, according to presence of topical therapy in the fellow eye

Presence of topical therapy in the fellow eye	Median IOP in the fellow eye, mmHg			Difference between preoperative IOP and IOP on the 1st postoperative day, P-value	Difference between preoperative IOP and IOP at one week after surgery, P-value
	preoperative	on the 1st postoperative day	at one week after surgery		
Fellow eyes without topical therapy (n=17)	16 (13-18)	13 (13-16)	14 (12-15)	0.72	0.08
Fellow eyes with topical therapy (n=170)	18 (15-21)	16 (14-20)	14 (12-17)	0.02*	<0.0001*

*P < .05
